# Ultradian oscillation in expression of four melatonin receptor subtype genes in the pineal gland of the grass puffer, a semilunar-synchronized spawner, under constant darkness

**DOI:** 10.3389/fnins.2015.00009

**Published:** 2015-01-30

**Authors:** Taro Ikegami, Yusuke Maruyama, Hiroyuki Doi, Atsuhiko Hattori, Hironori Ando

**Affiliations:** ^1^Department of Chemistry, Biology, and Marine Science, Faculty of Science, University of the RyukyusOkinawa, Japan; ^2^Department of Biology, College of Liberal Arts and Sciences, Tokyo Medical and Dental UniversityIchikawa, Japan; ^3^Shimonoseki Marine Science Museum “Kaikyokan,” Shimonoseki Academy of Marine ScienceYamaguchi, Japan; ^4^Sado Marine Biological Station, Faculty of Science, Niigata UniversitySado, Japan

**Keywords:** arylalkylamine *N*-acetyltransferase, circadian rhythm, circatidal rhythm, melatonin receptor, pineal gland, puffer, reproduction, ultradian rhythm

## Abstract

Melatonin receptor gene expression as well as melatonin synthesis and secretion activities were examined in the pineal gland of the grass puffer, which exhibits unique lunar/tidal cycle-synchronized mass spawing: spawning occurs before high tide on the day of spring tide during spawing season. Melatonin synthesizing activity was assessed by the abundance of arylalkylamine *N*-acetyltransferase 2 (AANAT2) mRNA. The amount of *aanat2* mRNA was low during light phase and initiated to increase after the light was turned off. The secretion of melatonin from primary pineal organ culture was stimulated after the light was turned off and ceased immediately after the light was turned on. The expression levels of four melatonin receptor subtype genes (*mel_*1a*_1.4*, *mel_*1a*_1.7*, *mel1b*, and *mel1c*) showed synchronous variations, and the levels tended to be high during the dark phase under light/dark conditions. These results suggest that the action of melatonin on the pineal gland is highly dependent on light and photoperiod, possibly with stronger action during night time. Under constant darkness, the expression of four melatonin receptor subtype genes showed unique ultradian oscillations with the period of 14.0–15.4 h, suggesting the presence of a circatidal oscillator in the pineal gland. The present results indicate that melatonin may serve local chronobiological functions in the pineal gland. These cyclic expressions of melatonin receptor genes in the pineal gland may be important in the control of the lunar/tidal cycle-synchronized mass spawning in the grass puffer.

## Introduction

Melatonin is produced mainly in the pineal gland and retina in fish, and its plasma concentration is higher during nighttime than daytime. This daily rhythm of circulating melatonin informs the organism about the time within a day, whereas the duration of the nocturnal elevation of melatonin that corresponds to photoperiod informs the organism about the season within a year (Reiter, 1993). Melatonin has been implicated in a wide variety of physiological and behavioral functions, such as circadian and seasonal rhythms, reproduction, growth, antioxidant action, immune response, sleep, feeding, locomotor activity, and depression (Pandi-Perumal et al., 2006; Falcón et al., 2010).

The actions of melatonin are mediated via melatonin receptors that belong to the G protein-coupled receptor superfamily (Reppert et al., 1996). In vertebrates, there are three types of melatonin receptors, Mel_1a_ (MT1), Mel_1b_ (MT2), and Mel_1c_. Mel_1a_ and Mel_1b_ have been identified in all vertebrate species investigated, whereas Mel_1c_ has been found only in non-mammalian species (Ebisawa et al., 1994; Reppert et al., 1995). Furthermore, two different subtypes of Mel_1a_ (Mel_1a_1.4 and Mel_1a_1.7) have been identified in zebrafish (Reppert et al., 1995), rainbow trout (Mazurais et al., 1999), goldfish (Ikegami et al., 2009a), grass puffer (Ikegami et al., 2009b), and mudskipper (Hong et al., 2014). Accordingly, phylogenetic analyses have shown that there are four subtypes of melatonin receptor genes in fish (Reppert et al., 1995).

Synchronous reproduction is crucial to reproductive success in most vertebrate species. The daily and seasonal control of reproduction involves cyclic and photoperiod-dependent changes in the activity of neurons secreting hypothalamic neuropeptides such as kisspeptin, gonadotropin-inhibitory hormone (GnIH) and gonadotropin-releasing hormone (GnRH) (Khan and Kauffman, 2012; Williams and Kriegsfeld, 2012; Simonneaux et al., 2013). These changes are brought in part by melatonin signals that transmit daily and photoperiodic information via melatonin receptors (Ubuka et al., 2005; Revel et al., 2008; Simonneaux et al., 2009; Yasuo et al., 2009). However, the mode of melatonin action on the reproductive neuroendocrine system remains to be determined.

The grass puffer (*Takifugu niphobles*) exhibits unique reproductive physiology and behavior that are synchronized with seasonal, lunar, and daily cycles. During the spawning season from spring to early summer, spawning occurs only during spring tide every 2 weeks (Yamahira, 2004; Motohashi et al., 2010; Ando et al., 2013). The fish aggregate at a certain seashore location for spawning that takes place in groups of 10–60 individuals, of which one is female. The fish usually aggregate at the spawning ground 2.5–3 h before high tide at night. Then, spawning starts 1.5–2 h before high tide and continues for 1 h during the rising tidal phase (Motohashi et al., 2010). Therefore, the timing of spawning is tightly connected with lunar and tidal rhythms as well as daily rhythm. Since we are aware of the time and place of the spawning, we can obtain spawning fish easily by dip net at the spawning bed. Thus, the grass puffer provides a unique animal model for studying the neuroendocrine mechanisms underlying the seasonal, lunar, and circadian control of reproduction.

Lunar-synchronized reproduction has been reported in a wide variety of organisms, particularly those living in shallow waters and reef areas. In these organisms, changes in moonlight and tide are considered to act as an environmental cue that entrains an internal clock for the synchronization of reproduction. However, the molecular mechanisms for lunar-synchronized spawning are poorly understood (Leatherland et al., 1992; Takemura et al., 2004a). In the golden rabbitfish, which spawns around the first quarter moon, the plasma levels of melatonin at midnight are higher on the day of new moon than full moon. This lunar phase-dependent variation in the plasma melatonin concentrations is critical for the occurrence of the lunar-synchronized spawning in the golden rabbitfish (Takemura et al., 2004b). The levels of melatonin receptor gene expression for *mt1* and *mel_*1c*_* showed variations depending on moonlight brightness in the pineal gland (Park et al., 2014). In addition, the levels of mudskipper *mel_*1a*_1.4* expression in the diencephalon show a lunar cycle-dependent variation with two peaks at the first and last lunar quarters when the fish spawns (Hong et al., 2014). These facts suggest that melatonin signals may play a key role in transmitting the photoperiodic information of moonlight to the reproductive neuroendocrine system in the hypothalamus.

Our previous studies on the grass puffer spawning rhythm also showed possible involvement of melatonin signals in the control of the semilunar-synchronized spawning. In the diencephalon, all four melatonin receptor subtype genes are synchronously expressed with daily and circadian variations under light/dark (LD) and constant darkness (DD) conditions, respectively (Ikegami et al., 2009b). In addition, not only kisspeptin (*kiss2*) and its receptor (*kiss2r*) genes but also LPXRFamide peptide gene (*lpxrfa*), fish ortholog of GnIH gene, and its receptor (*lpxrfa-r*) gene clearly showed daily and circadian oscillations in expression, and their expression patterns are almost synchronized with each other (Shahjahan et al., 2011; Ando et al., 2014). These results indicate that melatonin signals are highly dependent on light/dark cycle in the diencephalon, and melatonin may have an important role in the cyclic expressions of *kiss2*/*kiss2r* and *lpxrfa*/*lpxrfa-r* in the grass puffer.

In the present study, to further elucidate the role of melatonin signals in the control of the semilunar-synchronized spawning, daily and circadian oscillations in expression of the four melatonin receptor subtype genes were examined in the pineal gland of grass puffer. The pineal gland is one of the master clocks in fish (Falcón et al., 2009), and melatonin may have a local action on the pineal gland via melatonin receptor that leads to the production of the semilunar-synchronized spawning rhythm. In addition, daily and circadian changes in melatonin synthesis and secretion from the pineal gland was examined by cloning and expression analyses of gene encoding arylalkylamine-*N*-acetyltransferase (AANAT) 2, a rate-limiting enzyme in melatonin synthesis, and by measurement of melatonin secreted from primary pineal organ culture.

## Materials and methods

### Animals

Mature grass puffer of both sexes were caught by dip net at a spawning ground in Tomioka Bay, Kumamoto, Japan during spawning period in July and August 2009 and July 2010. They were transferred to the Fishery Research Laboratory Station, Kyushu University, Fukutsu, Japan and were kept in indoor tanks (500 l) with flow of seawater and under natural photoperiod (14L:10D, exact time of dawn and dusk were as follows: 5:20 and 19:30 in July 2009; 5:35 and 19:15 in August 2009; 5:25, and 19:25 in July 2010). The fish were fed commercial pellets equivalent to 1% of body weight (BW) at 9 a.m. daily. The experimental procedures followed the guidance approved by the Animal Care and Use Committees of Kyushu University, Fukuoka, Japan and Niigata University, Niigata, Japan.

### Sample collection

Daily variations of melatonin receptor and AANAT2 genes were examined by real-time PCR using the fish obtained in July 2009 (*n* = 56, 49 males, 50.0 ± 1.6 g in BW and 7 females, 49.9 ± 1.9 g in BW, July 18–19, age of the moon 26.0/middle tide, time of high tide 20:17, time of low tide 13:35) and July 2010 (*n* = 108, all males, 44.9 ± 0.7 g in BW, July 23–25, age of the moon 12.0/middle tide, time of high tide 21:33, time of low tide 15:17). The fish were transferred into indoor tanks (60 l) and acclimatized at 22°C for 6 days under natural photoperiod (14L:10D). After 3 days of fasting, the fish were anesthetized in 0.03% MS222, and killed by decapitation at 3 h intervals for 1 day at Zeitgeber time (ZT) 3, ZT6, ZT9, ZT12, ZT15, ZT18, ZT21, and ZT24 in 2009 (*n* = 7 for each time point) and for 2 days in 2010 (*n* = 6 for each time point). The whole brain including the pineal gland was removed and soaked in RNA*later* (Ambion, TX, USA) and was kept at 4°C for 1 day. The pineal gland was removed from the brain under a stereoscopic microscope and immediately frozen in liquid nitrogen and stored at −80°C.

For circadian variation, the fish obtained in August 2009 (*n* = 62, 48 males, 44.7 ± 1.4 g in BW and 14 females, 60.6 ± 3.3 g in BW, August 3–4, age of the moon 13.0/spring tide, time of high tide 21:37, time of low tide 15:22) and July 2010 (*n* = 136, 132 males, 45.2 ± 0.7 g in BW and 4 females, 47.9 ± 4.5 g in BW, July 7–9, age of the moon 25.0/middle tide, time of high tide 19:47, time of low tide 13:05) were acclimatized in the indoor tanks (60 l) for 6 days as described above. Then, the fish were left under DD condition without feeding for 3 days. The fish were anesthetized in 0.03% MS222 and killed by decapitation at 3 h intervals for 1 day at circadian time (CT) 3, CT6, CT9, CT12, CT15, CT18, CT21, and CT24 in 2009 (*n* = 6–7 for each time point) and for 2 days in 2010 (*n* = 8 for each time point). The whole brain including the pineal gland was removed under red dim light, and soaked in RNA*later* (Ambion, TX, USA). The pineal gland was collected as described above.

### Real–time PCR assay of melatonin receptor mRNAs

Real–time PCR assay was carried out as described previously (Ikegami et al., 2009b). Briefly, total RNA was extracted from the pineal gland and 200 ng of total RNA was used for synthesis of first strand cDNA by reverse transcription reaction using Multiscribe Reverse Transcriptase (Applied Biosystems, USA) according to the manufacturer's instruction. PCR reaction mixture (10 μl) contained 2 μl of sample cDNA, 0.2 μM of forward and reverse primers (Table 1) and 5 μl of SYBR Premix DimerEraser (Takara, Ohtsu, Japan). Amplification was carried out at 95°C for 30 s, followed by 40 cycles at 95°C for 5 s, 55°C for 30 s, and 72°C for 30 s. Specific amplification of each subtype cDNA was verified by melting curve analysis, gel electrophoresis of the product. The cross-reactivity with other subtype mRNAs in each assay was less than 0.29%. The slope and correlation coefficient (r) of the standard curve and the intra- and inter-assay coefficients of variation (CVs) in each assay are shown in Supplementary Table 1.

**Table 1:** **Primers used in the present study**.

	**Forward primer**	**Reverse primer**
**REAL-TIME PCR FOR MELATONIN RECEPTOR mRNAs**		
Mel1a1.4	GGCTCTTCACAGCCAGCTA	CGGAACTTGAAGACGATCAG
Mel1a1.7	TGGACTCGGTCTGAGCCAG	TCACGAAGCACCATGGTACAG
Mel1b	CCATAGATCCGTCCCACGTA	TGTTGAGCAGGCCATAGATG
Mel1c	ACGGAGACGTCGCGTTG	TCATGACGTTGGTCAACACG
**PARTIAL CLONING OF *aanat2 cDNA***		
AANAT2	TCCTCACCTCGACTCTGTC	TGGAAGTGCATGTTGGATATG
**REAL-TIME PCR FOR *aanat2 mRNA***		
AANAT2	ATCCACGTGTTGTCAGTACACC	AAGTCCTCGCAGATGAGCAG

### Partial cloning of *aanat2* and real–time PCR assay

Genomic DNA of grass puffer was prepared from blood using a Puregene DNA Purification Kit (Gentra, MN, USA). In order to design primers for cloning the grass puffer *aanat*2, the genome database of tiger puffer (http://uswest.ensembl.org/Takifugu_rubripes/Info/Index) were BLAST searched. There are three *aanat*s (*aanat1a*, *aanat1b*, and *aanat2*) in the tiger puffer genome, and all of them consist of 3 exons. PCR primers for the grass puffer *aanat2* were designed in the region from intron 1 to exon 3 (Table 1). PCR amplification using the grass puffer genomic DNA as template DNA was performed using a HotStar Taq Master Mix (Qiagen, Japan). Amplification was carried out at 95°C for 15 min, followed by 35 cycles of 94°C for 30 s, 53°C for 30 s and 72°C for 1 min, and finally by additional 10 min at 72°C. The PCR fragment of expected size was purified by a StrataPrep PCR Purification Kit (Stratagene, CA, USA) and cloned into a pGEM-T easy cloning vector (Promega, USA). The purified plasmid DNA was sequenced by a CEQ8800 DNA Analysis System (Beckman, Coulter).

Real–time PCR assay of *aanat2* mRNA was carried out as described above. PCR reaction mixture (10 μl) contained 2 μl of sample, 0.2 μM of forward and reverse primers (Table 1) and 5 μl of SYBR Premix DimerEraser (Takara, Ohtsu, Japan). Amplification was carried out at 95°C for 30 s, followed by 40 cycles at 95°C for 5 s, 55°C for 30 s, and 72°C for 30 s. Specific amplification of *aanat2* cDNA was verified by melting curve analysis and gel electrophoresis of the product. The slope, r, intra-assay CV, and inter-assay CV are shown in Supplementary Table 1.

### Primary organ culture of the pineal gland

The pineal gland was dissected out from adult grass puffer at ZT10, and were transferred to RPMI medium containing 20 mM HEPES, 9 mM sodium bicarbonate, penicillin (100 U/ml), streptomycin (100 U/ml) and fungizone (0.25 mg/ml), and pre-incubated at 20°C for 4 h. Two pineal glands were placed on sterile glass wool in a superfusion chamber (5 mm in diameter, 20 mm in height). The medium was superfused to keep the volume in the chamber 0.2 ml. The entire apparatus including the culture medium stock and the culture chamber was placed in an incubator at 20°C. A white fluorescent light was set in the incubator, and light intensity at the surface of the incubation chamber was approximately 1400 lux. The pineal glands were maintained for 1 day under LD condition (14L:10D) and then the light was turned off to keep them under DD condition for 31 h. The culture medium was continuously pumped at a rate of 1 ml/h and the perfusate was collected hourly by a fraction collector (FRAC-200, Amersham Biosciences). This primary culture experiment using two pineal glands was repeated four times.

### Melatonin measurement

The melatonin concentrations in the culture medium were measured as described previously (Itoh et al., 1997). Melatonin was extracted from 0.3 ml of perfusate by mixing with chloroform (4 ml) and distilled water (1 ml). After centrifugation at 3000 rpm for 1 min, the aqueous phase was discarded and the organic phase was evaporated with a vacuum evaporator. The extracts were redissolved in 300 μl of HPLC mobile phase solution consisting of 50 mM ammonium acetate and 30% methanol (vol/vol), adjusted to pH 4.8 with acetic acid. After centrifugation at 500 × g for 1 min at room temperature, the supernatant was filtrated through a Millex LH 0.45 μm filter unit (Millipore, Bedford, MA, USA) and subjected to chromatography using a CAPCELL PAC C18 MGII 5 μm column (4.6 × 250 mm) (Shiseido, Tokyo, Japan) and RF-10AXL fluorometric detector (Shimadzu, Kyoto, Japan). The detector was operated at an excitation wavelength of 280 nm and an emission wavelength of 340 nm. All separations were performed isocratically at mobile phase flow rate of 0.8 ml/min and 40°C. The fraction corresponding to the authentic melatonin peak was collected. Peaks were identified by retention time and melatonin was quantified by peak area. The limit of sensitivity of the assay was as low as 1 pg for a 2:1 signal-to-noise ratio. Intra- and inter-assay CVs were 0.52% (*n* = 3) and 1.15% (*n* = 5), respectively. Melatonin was obtained from Sigma (St. Louis, MO, USA).

### Statistical analysis

The amounts of melatonin receptor and *aanat2* mRNAs are expressed as means ± SEM. Data were analyzed by one-way analysis of variance (ANOVA) followed by Tukey's test or Games–Howell's multiple comparisons test to assess statistically significant differences among the different time points in the daily and circadian variation experiments. The periodicity of daily and circadian variations was calculated with COSINOR (http://www.circadian.org/softwar.html).

## Results

### Daily and circadian variations in melatonin secretion from the pineal gland

**Figure 1:**
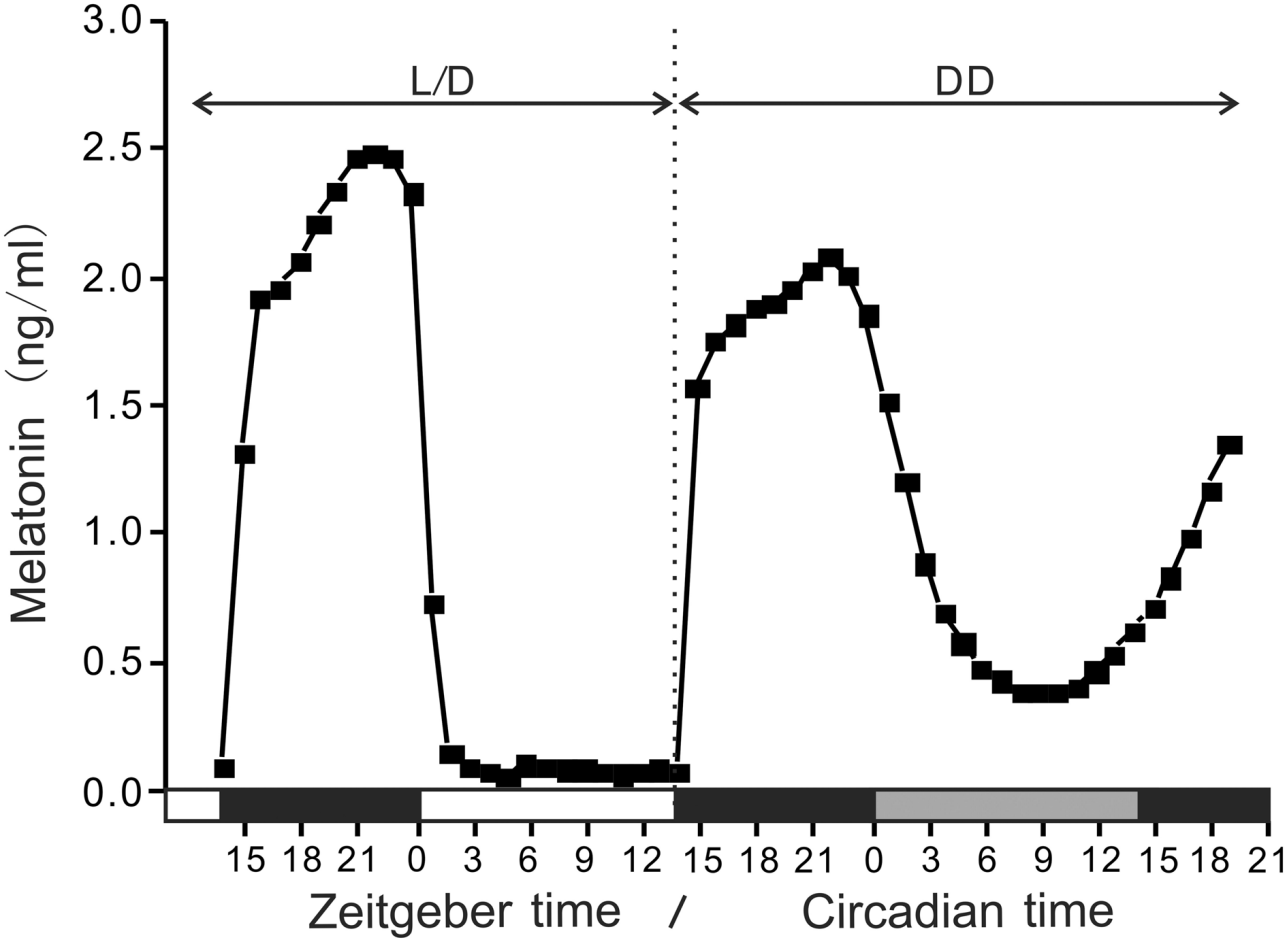
**Daily and circadian changes in melatonin secretion from the pineal gland *in vitro***. Two pineal glands were placed in a superfusion chamber and maintained for 1 day under LD condition (14L:10D, 1400 lux at the surface of the incubation chamber) and then the light was turned off to keep them under DD condition for 31 h. The culture medium was collected hourly by a fraction collector, and the concentrations of melatonin in the perfusate were determined by high performance liquid chromatography. This primary culture experiment using two pineal glands was repeated four times and a representative profile is shown.

The secretion pattern of melatonin from the pineal gland was examined using the primary organ culture system. Under LD conditions, the medium melatonin concentrations significantly increased during dark phase and quickly dropped after exposure to light (Figure 1). During the light phase, the medium melatonin levels remained at almost zero. This daily change was repeated at least for 3 days under the LD conditions in this culture system (data not shown). Under DD conditions, the medium melatonin levels showed a circadian variation with lowest levels at CT9 (middle of subjective light phase), but the levels were significantly higher than that at ZT9 (0.07 ± 0.01 ng/ml at ZT9 vs. 0.68 ± 0.05 ng/ml at CT9, *n* = 4, *p* < 0.001 by *t*-test). The levels were initiated to increase at the end of subjective light phase. The COSINOR analysis revealed a significant circadian rhythm with 23.7 h period (*p* < 0.001).

### Daily and circadian oscillations in expression of *aanat2* in the pineal gland

Partial DNA sequence determined for the grass puffer *aanat2* was 454 bp including exons 2–3 (Accession No. LC010911). The coding region of 383 bp encodes a predicted AANAT2 protein that contains conserved regions including C/c-1, D/c-1, D/c-2, and motifs A and B (Supplementary Figure 1). The nucleotide sequence similarity of *aanat2* between grass puffer and tiger puffer is 98.2%.

**Figure 2:**
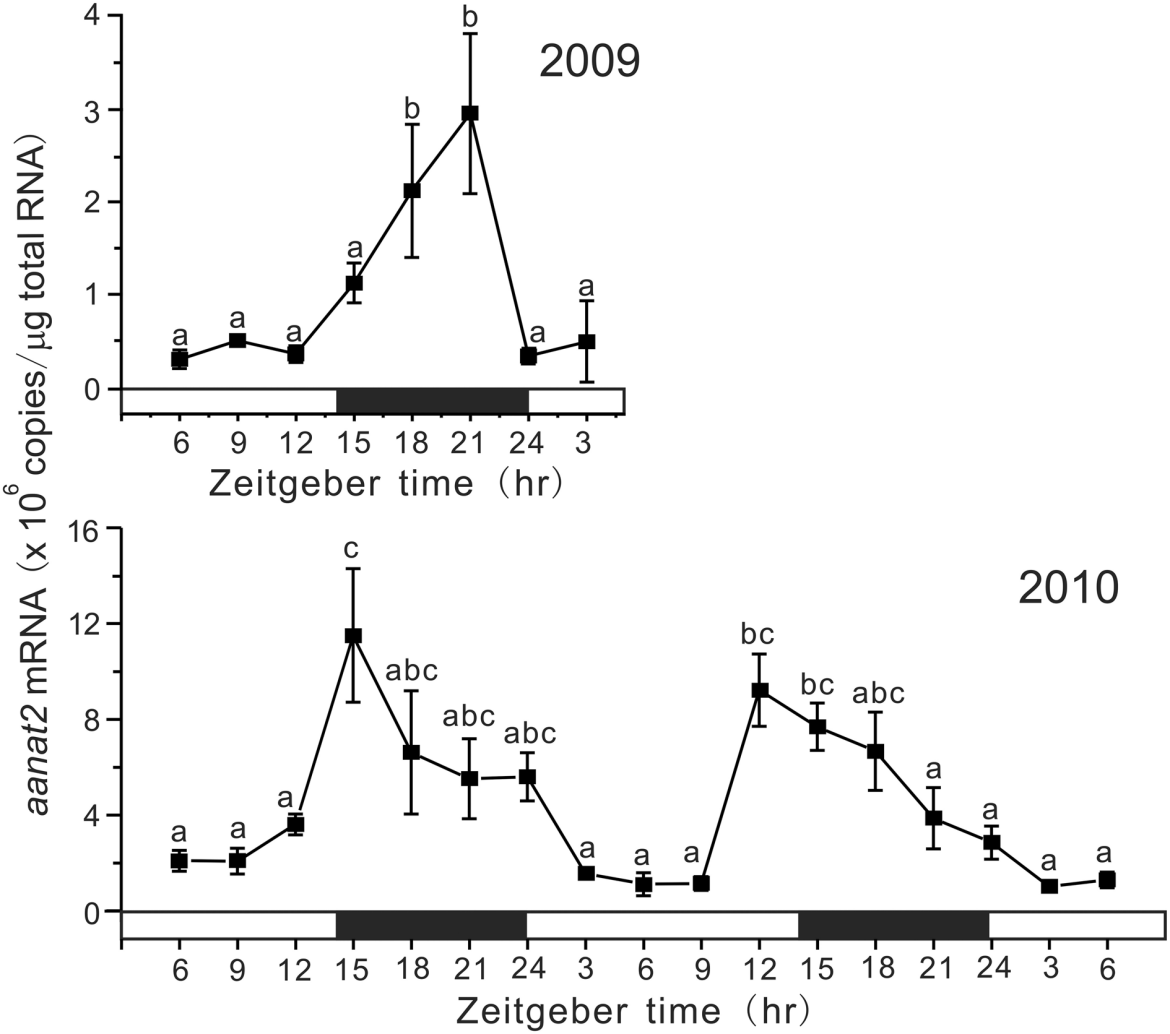
**Daily variations in the amounts of *aanat2* mRNA in the pineal gland**. The fish were kept under natural photoperiod (14L:10D), and the brain samples were taken at 3 h intervals for 1 day at ZT3, ZT6, ZT9, ZT12, ZT15, ZT18, ZT21, and ZT24 in 2009 (*n* = 7 for each time point) and for 2 days in 2010 (*n* = 6 for each time point). Values accompanied by different letters are statistically significantly different (*p* < 0.05).

Under LD conditions, the absolute amounts of *aanat2* mRNA were low during the light phase and significantly increased at the end of the light phase or during the dark phase, although the peak levels and positions were different (3 × 10^6^ copies/μg RNA at ZT21 in 2009 and approximately 11 × 10^6^ copies/μg RNA at ZT12-15 in 2010 (Figure 2). The COSINOR analyses for the variations in 2009 and 2010 revealed significant daily rhythms with 19.3 h period (*p* < 0.05) and 21.0 h period (*p* < 0.001), respectively.

**Figure 3:**
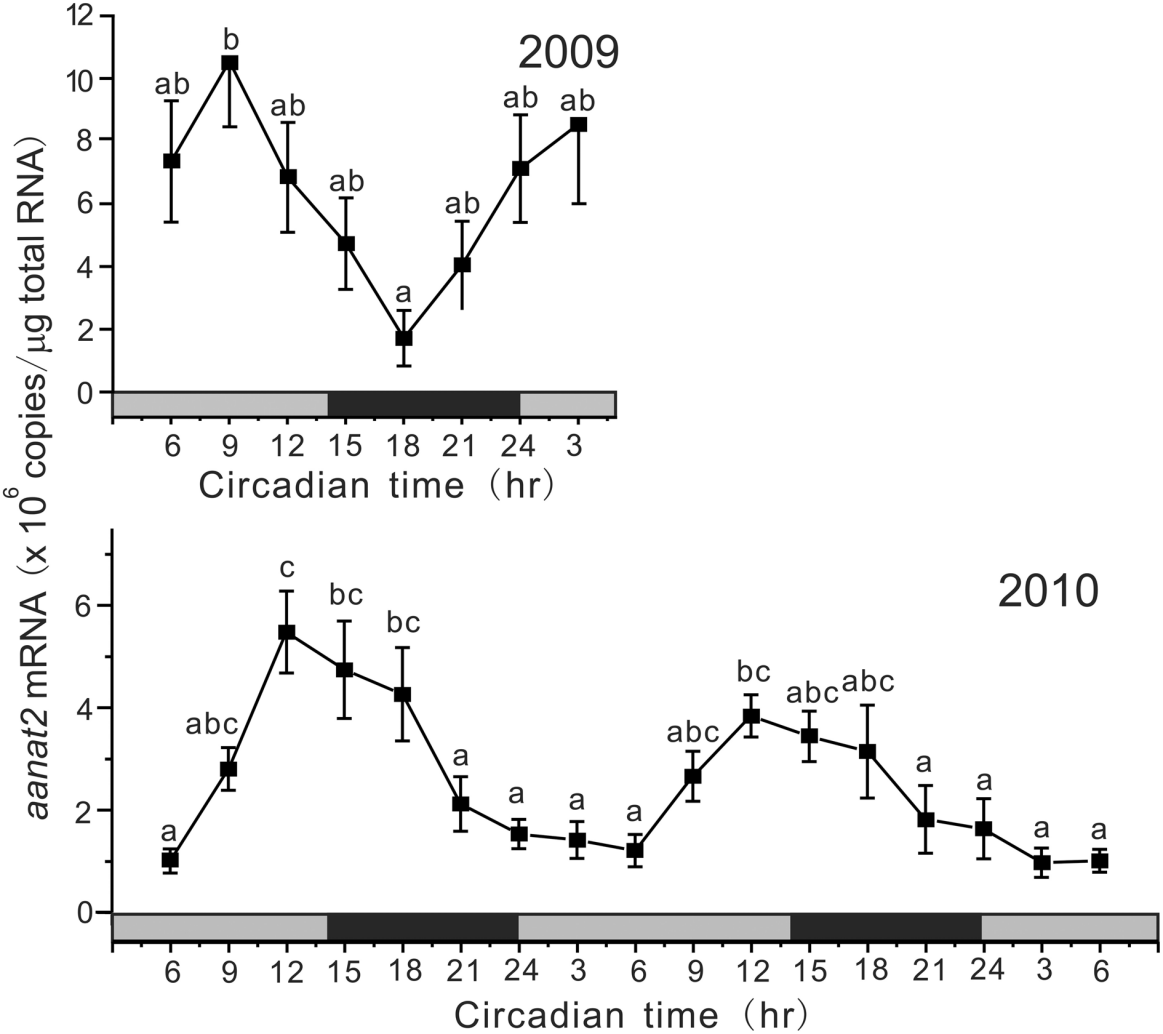
**Circadian variations in the amounts of *aanat2* mRNA in the pineal gland**. The fish were kept under constant darkness condition for 3 days, and then the brain samples were taken under red dim light at 3 h intervals for 1 day at CT3, CT6, CT9, CT12, CT15, CT18, CT21, and CT24 in 2009 (*n* = 6–7 for each time point) and for 2 days in 2010 (*n* = 8 for each time point). Values accompanied by different letters are statistically significantly different (*p* < 0.05).

Under DD conditions, *aanat2* showed circadian oscillation, but the profiles were different between 2009 and 2010 (Figure 3). In 2009, the mRNA levels showed a peak at CT9 and the lowest level at CT18 with a significant circadian rhythm with 18.3 h period (*p* < 0.01). In 2010, *aanat2* showed a somewhat different circadian oscillation to that in 2009: the low levels of mRNA continued for longer period from CT21 to CT6 with a significant circadian rhythm with 24.0 h period (*p* < 0.001).

### Daily and ultradian oscillations in expression of four melatonin receptor subtype genes in the pineal gland

**Figure 4:**
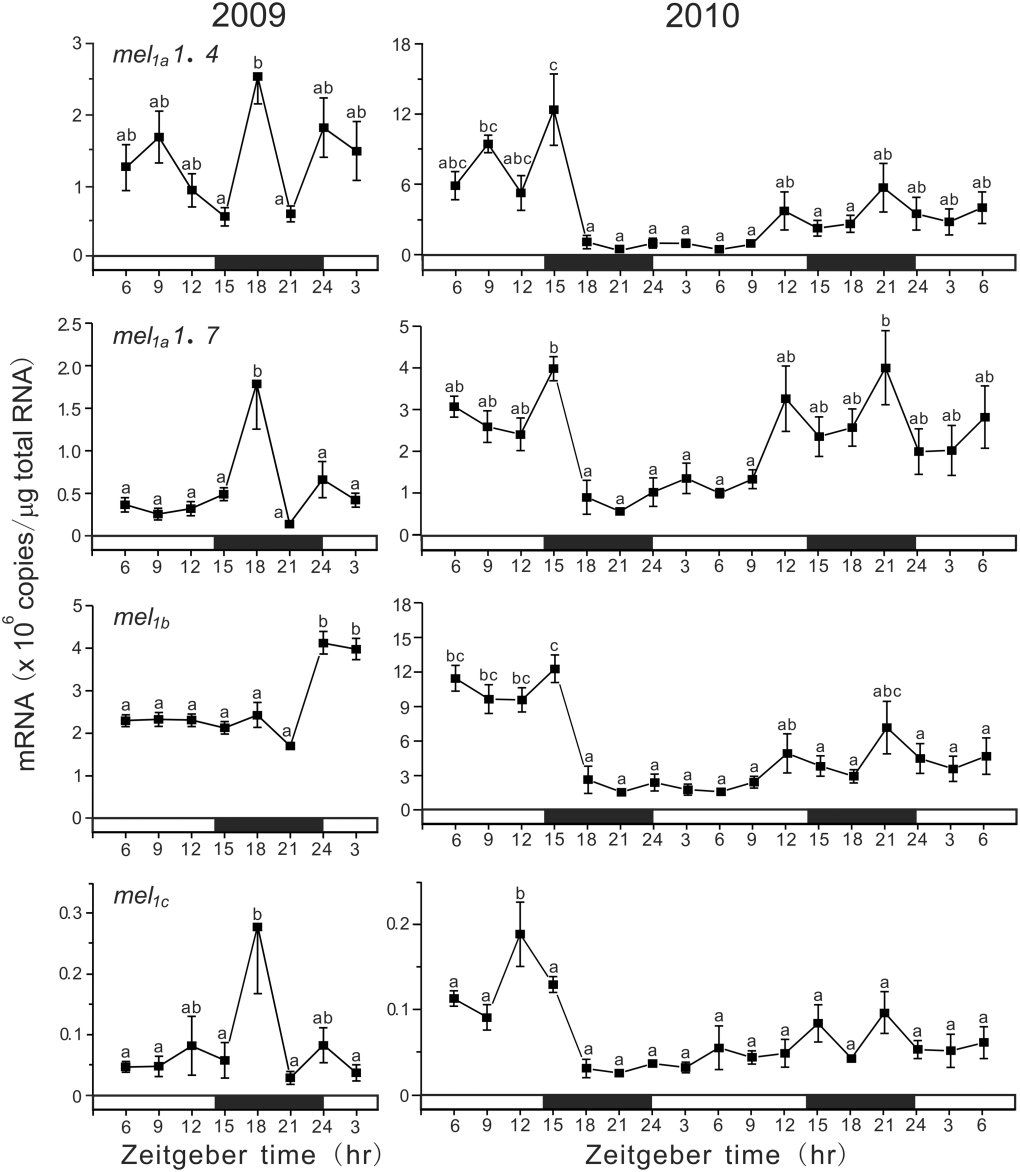
**Daily variations in the amounts of four melatonin receptor subtype mRNAs in the pineal gland**. The fish were kept under natural photoperiod (14L:10D), and the brain samples were taken at 3 h intervals for 1 day at ZT3, ZT6, ZT9, ZT12, ZT15, ZT18, ZT21, and ZT24 in 2009 (*n* = 7 for each time point) and for 2 days in 2010 (*n* = 6 for each time point). Values accompanied by different letters are statistically significantly different (*p* < 0.05).

In the pineal gland, the absolute amounts of melatonin receptor subtype mRNAs were comparable for *mel_*1a*_1.4*, *mel_*1a*_1.7*, and *mel_*1b*_* with highest levels of *mel_*1b*_* mRNA (Figure 4). The amounts of *mel_*1c*_* mRNA were as low as approximately one thirtieth those of *mel_*1b*_* mRNA. In 2009 under LD conditions, the mRNA amounts of *mel_*1a*_1.4*, *mel_*1a*_1.7*, and *mel_*1c*_* showed a synchronous daily variation with a sharp peak at ZT18, whereas *mel_*1b*_* showed a somewhat arrhythmic expression pattern. In 2010, the expression levels of four melatonin receptor subtype genes showed a synchronous variation for 2 days. The levels tended to be high during the dark phase, although these changes were less cyclic.

**Figure 5:**
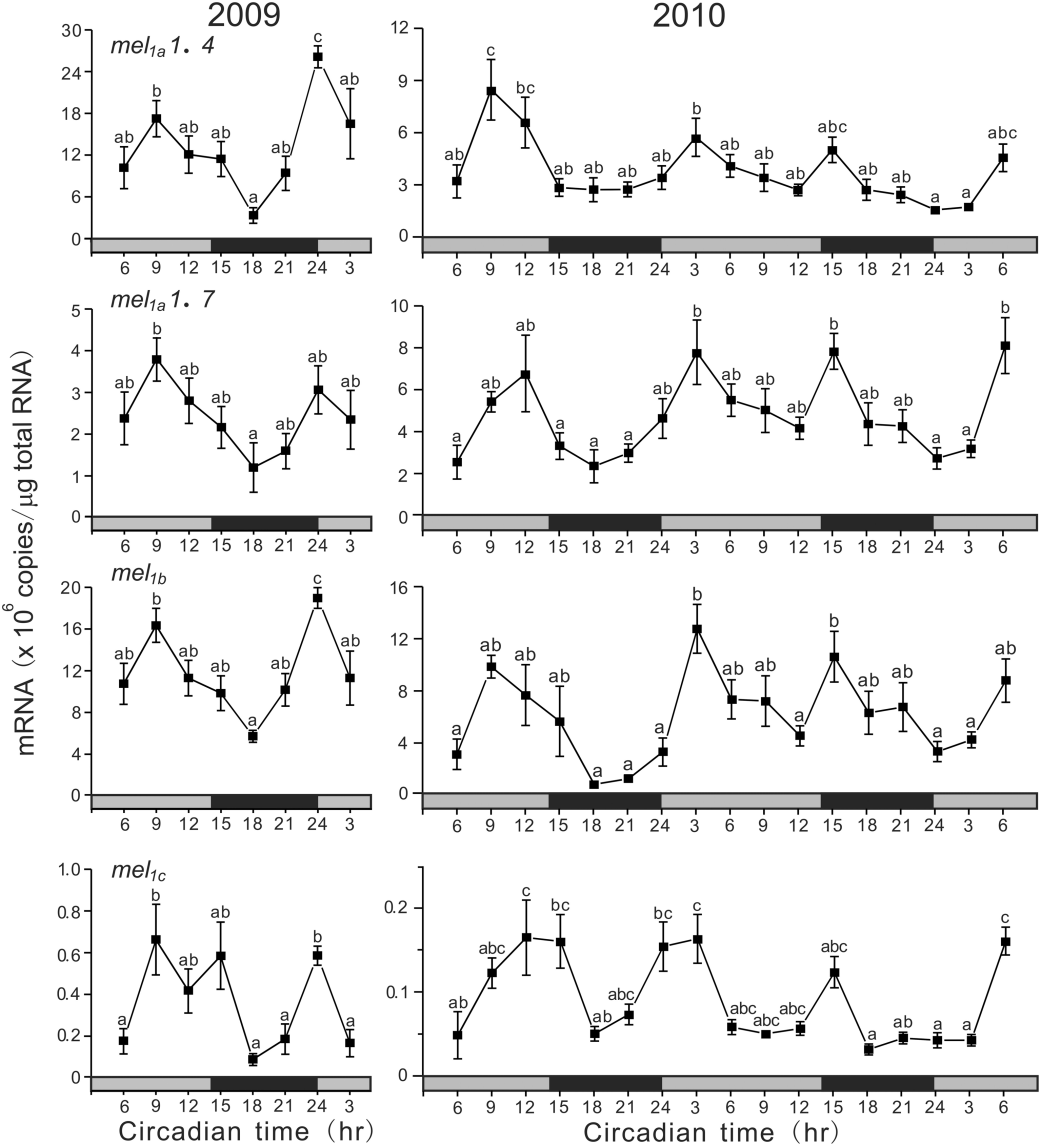
**Ultradian variations in the amounts of four melatonin receptor subtype mRNAs in the pineal gland**. The fish were kept under constant darkness condition for 3 days, and then the brain samples were taken under red dim light at 3 h intervals for 1 day at CT3, CT6, CT9, CT12, CT15, CT18, CT21, and CT24 in 2009 (*n* = 6–7 for each time point) and for 2 days in 2010 (*n* = 8 for each time point). Values accompanied by different letters are statistically significantly different (*p* < 0.05).

Under DD conditions, all subtype genes showed a synchronous ultradian oscillation in expression (Figure 5). In 2009, all four subtype genes showed synchronous variations with two peaks at CT9 (middle of subjective light phase) and CT24 (start of subjective light phase). The COSINOR analyses revealed significant circadian rhythms with 14.6 h period for *mel_*1a*_1.4* (*p* < 0.05), 15.4 h period for *mel_*1a*_1.7* (*p* < 0.05), and 14.5 h period for *mel_*1b*_* (*p* < 0.05). In 2010, the four subtype genes also exhibited ultradian oscillation in expression continuously for 2 days. The COSINOR analyses revealed significant circadian rhythms with 15.4 h period for *mel_*1a*_1.4* (*p* < 0.01), 14.5 h period for *mel_*1a*_1.7* (*p* < 0.001), 14.9 h period for *mel_*1b*_* (*p* < 0.01), and 14.0 h period for *mel_*1c*_* (*p* < 0.001).

## Discussion

In the present study, melatonin receptor gene expression as well as melatonin synthesis and secretion activites were examined in the pineal gland of grass puffer, a semilunar-synchronized spawner. Melatonin synthesizing activity was assessed by the abundance of *aanat2* mRNA, which encodes a rate-limiting enzyme in the conversion of serotonin to melatonin. The amount of *aanat2* mRNA were low during light phase and was initiated to increase after the light was turned off. The secretion of melatonin from the pineal organ culture was drastically stimulated after the light was turned off and ceased immediately after the light turned on. Accordingly, the melatonin synthesis and secretion is certainly dependent on light, and melatonin is secreted only during dark phase. On the other hand, four melatonin receptor subtype genes mostly showed synchronous expression with a peak during dark phase. These results suggest that the action of melatonin on the pineal gland is highly dependent on light and photoperiod, possibly with stronger action during night time. Interestingly, the four melatonin receptor genes showed unique ultradian oscillations with the period of 14.0–15.4 h under DD conditions. To our knowledge, this is the first description of ultradian oscillation in melatonin receptor gene expression under DD conditions. This unique ultradian expression of melatonin receptor genes may be involved in the control of the semilunar-synchronized spawning rhythm in the grass puffer.

In this study, we identified three *aanats* in the tiger puffer genome, and a partial nucleotide sequence of the grass puffer *aanat2* was determined. Two *aanat* genes, *aanat1* and *aanat2*, have been identified in teleosts (Coon et al., 1999; Benyassi et al., 2000; Shi et al., 2004; Zilberman-Peled et al., 2004; Vuilleumier et al., 2007). In addition, two subtypes of *aanat1* genes, *aanat1a* and *aanat1b*, have been predicted in the genomes of tiger puffer and medaka (Falcón et al., 2009), and their cDNAs were isolated from the Senegalese sole retina (Isorna et al., 2011). *aanat1* is mainly expressed in the retina, whereas *aanat2* is expressed exclusively in the pineal gland. The deduced grass puffer AANAT2 contains plausible arylalkylamine binding domains (C/c-1, D/c-1, and D/c-2) (Klein et al., 1997), and highly conserved regions of *N*-acetyltransferase superfamily (motifs A and B). Site directed mutagenesis in yeast MAK3 and human spermidine/spermine *N*-acetyltransferases revealed that motifs A and B are important to maintain enzyme activities (Tercero et al., 1992; Coleman et al., 1996). The nocturnal expression of the grass puffer *aanat2* was apparent in both 2009 and 2010 (Figure 2), and this is well-consistent with the nocturnal secretion of melatonin *in vitro* (Figure 1). Under DD conditions, the grass puffer *aanat2* exhibited cyclic expression patterns with a peak at CT9 in 2009 and at CT12 or CT15 in 2010. The profiles of *aanat2* expression were somewhat different between 2009 and 2010 possibly due to variation in natural light conditions in the 2 years. It is assumed that the grass puffer *aanat2* expression shows daily and circadian oscillation through regulation by the internal circadian clock, as reported in other species (Foulkes et al., 1997; Coon et al., 1999; Kashiwagi et al., 2013).

Melatonin secreted from the pineal gland has been shown to be involved in the control of daily and seasonal rhythms in many physiological and behavioral functions through melatonin receptors. The present study demonstrated cyclic changes in expression of all four melatonin receptor genes in the pineal gland, and their expression patterns are mostly synchronized. It is thus conceivable that melatonin may serve local functions in the pineal gland that are most probably connected to rhythmic processes. In mammals, melatonin has been shown to play a role in resetting the circadian pacemaker activity in the suprachiasmatic nucleus (SCN) via MT2 (Liu et al., 1997). Melatonin directly influences on the electrical and metabolic activities of the SCN, resulting in the phase-shifting effect and also a significant increase in amplitude of the oscillations (Pévet et al., 2002). Since in fish the master circadian clock is considered to be located in the pineal organ in addition to eyes and probably hypothalamus (Falcón et al., 2009), the pineal melatonin may exhibit a local action on the activity of circadian clock. The expression of the four melatonin receptor genes tended to increase during the dark phase (Figure 4), indicating that melatonin's chronobiotic effect is certainly dependent on light and time. Taken together with the nocturnal melatonin secretion, the effect may be more drastic during the dark phase.

The daily oscillation in expression of melatonin receptor genes has been reported in the brain of various fish species (Park et al., 2006, 2007a,b; Ikegami et al., 2009a,b; Confente et al., 2010; Chai et al., 2013). In the diencephalon of grass puffer, all four subtype genes showed a peak at ZT15 under LD conditions like in the pineal gland (Ikegami et al., 2009b). Similarly, ZT-dependent fluctuations in expression of the four melatonin receptor genes were observed in the grass puffer retina and optic tectum, although in some cases including the pineal glands in 2010, the mRNA amounts showed arrhythmic variations. This might be due to effects on melatonin receptor gene expression of some other environmental and internal conditions, such as water temperature, nutrition, sexual maturation, and infection (immune response) (Pandi-Perumal et al., 2006; Falcón et al., 2010). Nevertheless, it is of considerable interest to note that in the grass puffer diencephalon, *kiss2*/*kiss2r* and *lpxrfa*/*lpxrfa-r* also showed daily and mostly synchronized oscillations (Shahjahan et al., 2011; Ando et al., 2014). These results suggest that the reproductive neuroendocrine activity may be cyclic within a day under the control of melatonin signals directly or indirectly via circadian clock in the pineal gland (Ando et al., 2013, 2014). The involvement of MT1 in the control of *gnrh1* expression through *kiss2* was reported in the orange-spotted grouper (Chai et al., 2013).

Recently, studies on melatonin receptor gene expression in lunar-dependent spawner indicated that their expressions are dependent on the lunar phase, e.g., *mt1* and *mel_*1c*_* in the pineal gland of golden rabbitfish (Park et al., 2014) and *mel_*1a*_1.4* in the diencephalon of mudskipper (Hong et al., 2014). Thus, the melatonin signals may play a key role in transmitting the photoperiodic information of moonlight to the reproductive neuroendocrine system. Taking together with the clear daily and circadian expressions of kisspeptin and LPXRFa genes in the grass puffer (Shahjahan et al., 2011; Ando et al., 2014), it is possible that the lunar cycle-dependent changes in the melatonin/melatonin receptor levels may produce lunar-related oscillations of kisspeptin and LPXRFa gene expressions in addition to the daily oscillations. So far, the plasma melatonin levels could not be determined in the grass puffer due to the presence of interfering material in the assay, and monthly variations of the plasma melatonin levels and melatonin receptor gene expression are currently under investigation.

Under DD conditions, all four melatonin receptor genes showed ultradian oscillations with the period of 14.0–15.4 h in both 2009 and 2010 (Figure 5). This unique ultradian rhythm in melatonin receptor gene expression leads us to speculate that this rhythm might be related to circatidal rhythm, the period of which is 12.4 h, and there must be a circasemidian clock in the pineal gland of the grass puffer in addition to the circadian clock. Circatidal rhythms have been reported in behavioral and physiological activities of various marine aminals, for example in crab (Saigusa, 2002; Chabot et al., 2004), cumacean (Akiyama, 2004), cricket (Satoh et al., 2008) and ragworm (Last et al., 2009). On the other hand, circasemidian rhythms have been reported in humans (Wan et al., 1992; Hayashi et al., 2002; Tarquini et al., 2005). Interestingly, the combination of a circatidal oscillator with a circadian oscillator can produce circasemilunar oscillations which enable an animal to synchronize its rhythms with the environmental situation that reoccurs every 15 days at the same time of day (Bünning and Müller, 1961). It should be of considerable interest and importance to determine if this ultradian rhythm of melatonin receptor gene expression is entrained with the tidal changes when the fish are reared under such situation. If so, the pineal gland would be able to produce semilunar oscillations of melatonin signals without changes in moonlight. Alternatively, there may be a circasemilunar oscillator that can be entrained with moonlight (Neumann, 1989). Although further studies will be needed to examine which hypothesis is correct for the semilunar spawning rhythm of grass puffer, the present results indicate that the grass puffer provides a unique and useful animal model for studying the molecular and physiological mechanisms underlying the semilunar-synchronized biological rhythm.

In conclusion, in the grass puffer pineal gland, the activity of melatonin synthesis and secretion was solely dependent on light and time, and melatonin is secreted only during dark phase. Four melatonin receptor subtype genes mostly showed synchronous expression with a peak during dark phase, suggesting that melatonin may serve local chronobiotic functions in the pineal gland that might be influenced by moonlight. Moreover, the four melatonin receptor genes showed unique ultradian oscillations under DD conditions with the period of 14.0–15.4 h, suggesting the presence of a circasemidian oscillator. Taken together, the cyclic expressions of melatonin receptor genes may be important in the control of the semilunar-synchronized spawning rhythm in the grass puffer.

### Conflict of interest statement

The authors declare that the research was conducted in the absence of any commercial or financial relationships that could be construed as a potential conflict of interest.
