# Supplementary figures and images for: Ultradian oscillation in expression of four melatonin receptor subtype genes in the pineal gland of the grass puffer, a semilunar-synchronized spawner, under constant darkness
Source: Front Neurosci. 2015 Jan 30;9:9. doi: 10.3389/fnins.2015.00009 (PMC4311631; doi:10.3389/fnins.2015.00009)

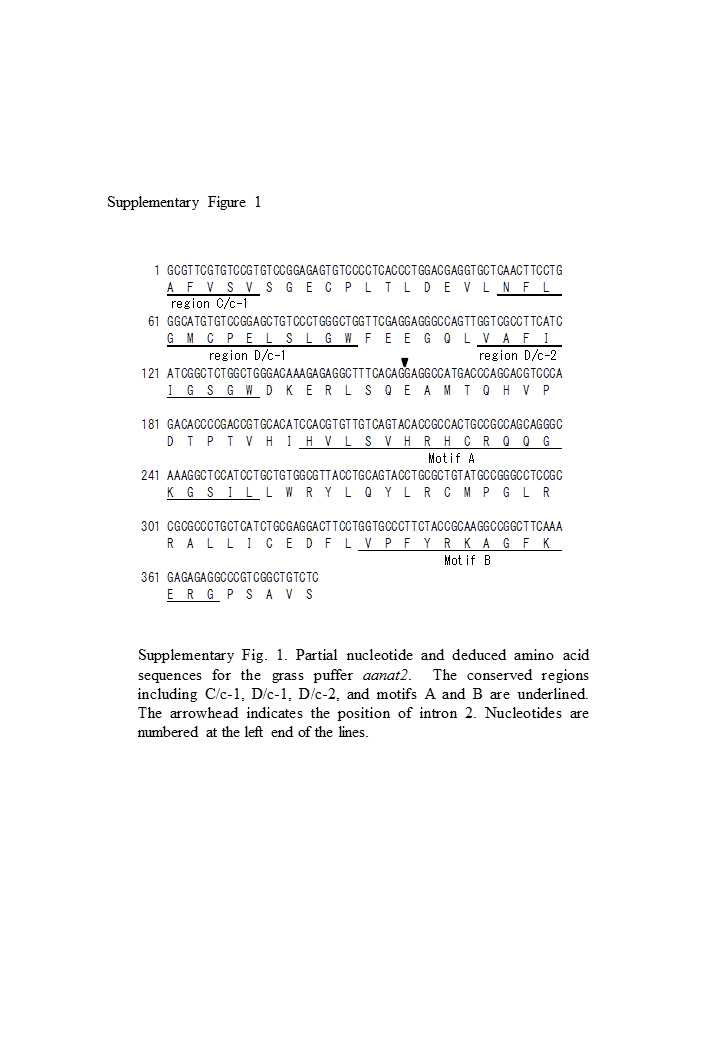

Supplement: Supplementary file 2 [file Image1.TIF]
